# Can a Purposeful Walk Intervention with a Distance Goal Using an Activity Monitor Improve Individuals’ Daily Activity and Function Post Total Hip Replacement Surgery. A Randomized Pilot Trial

**DOI:** 10.34133/cbsystems.0069

**Published:** 2023-11-30

**Authors:** Shayan Bahadori, Jonathan Mark Williams, Sarah Collard, Ian Swain

**Affiliations:** ^1^ Orthopaedic Research Institute, Bournemouth University, Bournemouth, Dorset, UK.; ^2^ Faculty of Health and Social Sciences, Bournemouth University, Bournemouth, Dorset, UK.; ^3^ Faculty of Science and Technology, Bournemouth University, Poole, Dorset, UK.

## Abstract

Individuals have increasingly high expectations of return to activity following total hip replacement (THR) surgery. The current literature demonstrates marked improvements in pain following THR. However, there is limited evidence showing objective improvement in daily activity. This randomized pilot trial aimed to determine the effect of an intervention where outdoor walking distance is used as a goal to increase daily activity of older adults using a commercial activity monitor at 3 to 6 months post THR. Findings suggested that the participants in the intervention group had higher activity levels after THR, compared to those in the control group. The Cohen’s effect sizes were larger for the changes in the gait, Hip Disability and Osteoarthritis Outcome Score, and Psychosocial Impact of Assistive Devices Scale data in the intervention group in contrast to the control group. However, further research with a larger sample size is required to provide tangible evidence on the significance of the effect of the purposeful walk compared to step count.

## Introduction

Total hip replacement (THR) is one of the most common and successful orthopedic operations worldwide [1,2] that offers pain relief even at week 1 postsurgery [3–5]. However, a recent report [6] suggested that the aim should not only be to improve pain but also lead to improving physical activity. This activity should preferably meet the recommended daily activity levels (at least 150 to 300 min of moderate-intensity physical activity per week) by the World Health Organization [7].

Despite the recommendations and evidence showing the benefit of physical activity, previous research has reported that most individuals undergoing THR are not physically active enough after their surgery [8]. Recent studies [9–12] monitored the recovery of individual post-THR surgery, and they found that the number of steps decreases and does not reach the same level as before surgery even at 24 months postsurgery period. Furthermore, physical activity greatly benefits human movement. For THR patients, increased physical activity links to better biomechanics during walking and daily tasks [13,14]. Regular exercise enhances muscle strength, joint flexibility, and cardiovascular fitness, leading to smoother movement [15]. THR patients benefit from improved joint range, muscle strength, and coordination through physical activity [14]. This aids in restoring optimal biomechanics and overall mobility.

Activity monitors have been extensively used as an incentive to encourage people in the wider population to become more active through walking [16]). For example, Simonsick and Guralnik [17] and Geurts and Van Geel [18] carried out large longitudinal studies in a group of female older adults and individual with multiple sclerosis respectively and found that the activity monitor increases walking distance among their cohorts. These studies utilized different types of activity monitors, but the major incentive for such enhancement were the targets that were set for the individual throughout the study. However, when it comes to the THR cohort, the evidence of distance-based interventions is limited, in particular when it comes to outdoor walking [19–22]. The focus of current studies has been merely on monitoring or enhancing the amount of walking using the step count parameter. This is a shortcoming because a recognized technical problem with the activity monitors is their diminishing accuracy in step counting associated with decreased walking speed [23] which is often a gait characteristic associated with people after THR operation. Additionally, there is currently a lack of attention for personalized plans in the postoperative period which is against the desire of individuals undergoing THR surgery [24]. Further evidence also suggest that individuals undergoing THR surgery are interested and receptive of wearable technologies and, in particular, enjoy the outdoor elements where sensors such as Global Positioning System (GPS) technology are used to track their daily outdoor activities [6,19,22,24,25].

This study aims to determine the effect of an intervention where an outdoor walking distance is used as a goal to increase daily walking activity, using a commercially available activity monitor, in people after THR 3 to 6 months post THR surgery. Throughout this protocol, we will refer to the outdoor walk that is recorded with a GPS sensor as a “purposeful walk”.

## Methods

### Trial design

This was an investigator-initiated, single-center randomized pilot trial with full ethical approval granted by the Bournemouth University Research Ethics Committee (ref: 45499) and prepared in accordance with CONSORT guidelines for reporting randomized pilot studies [26]. A CONSORT checklist of information is included in the Supplementary Materials (Appendix S1).

### Participants

Table 1 provides full eligibility criteria for the participants in the study. Participants were all recruited through publicizing tools such as Twitter posts and posters shared on the University channels (Bournemouth University research blogs, the Public Involvement in Education and Research group, University of Third Age, and communities of older adults [e.g., local indoor bowling clubs]). Those interested in the study contacted the lead researcher, were provided with an information sheet, and, to comply with Good Clinical Practice guidelines [27], were given 48 hours to consider participating.

**Table 1.** Eligibility criteria

Inclusion criteria	• Male and female, aged 60 years and over • 3 to 6 mo post unilateral total hip replacement surgery for osteoarthritis • Can provide verbal confirmation that they have been discharged from their surgical care • Capable of independent walking • Capable of completing the activity diary independently • Have access to a smartphone or computer • Willing to complete the trial protocol
Exclusion criteria	• Unable to provide informed consent • Unable to complete follow-up (insufficient English, lives overseas, unable to return easily) • Not physically able to use Grail gait lab • Systematic disease affecting walking ability (chronic obstructive pulmonary disease (COPD), congestive heart failure (CHF), chronic kidney disease (CKD), Parkinson’s disease, cerebral palsy, multiple sclerosis, etc.) • Requiring revision hip replacement • Previous hip replacement (resurfacing or THR) on the contralateral side • Known metastatic tumor involving the hip.

### Setting

**Fig. 1.**
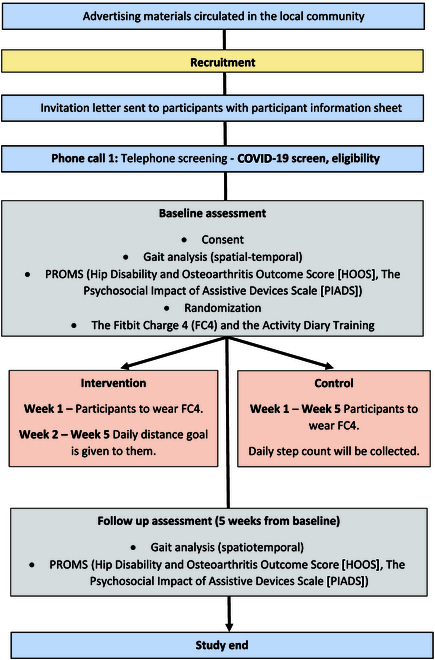
Study flow chart and group design.

The study was carried out at the Orthopaedic Research Institute at Bournemouth University. Following informed consent, participants were assigned to either the intervention or the control group. Details on randomization process is explained in Randomization. Figure 1 outlines the study flow.

### Intervention group

The purposeful walking intervention group in this study was monitored using the Fitbit Charge 4 (FC4) (Fitbit.com, Google Device, USA) activity monitor. Participants wore the FC4 activity monitor for 5 weeks in total. In the first week, participants wore their FC4 activity monitor in order to understand the participant’s postsurgical purposeful walking distances. In week 2, a target distance was calculated to increase the weekly walking distance by 10% and was divided by 7 to calculate a daily distance for that week. In the weeks thereafter, if participants achieved their target, a new purposeful distance target was calculated to increase the participant’s walking distance by a factor of 10% from the previous target. If the participant did not meet their target, the daily distance goal they were assigned the previous week remained in place. Participants were contacted through the FC4 Fitbit app on a weekly basis throughout the study and were given their daily goals for the upcoming week. The FC4 activity monitor was worn on the wrist of the nondominant hand continuously during the study period. Participants were shown how to charge and operate the FC4 activity monitor and were given a copy of a simple instruction manual to take with them.

### Control group

Participants in the control group wore the FC4 activity monitor for 5 weeks in total but were not given any weekly distance target and were asked to report their daily number of steps. The benefits of distance-based walking, in contrast to step count, have already shown benefits in reducing cardiovascular disease [28], but to our knowledge, this is the first study to examine the efficacy of outdoor distance-based walking in a group of THR patients. Furthermore, it cannot be guaranteed that the control group will walk outside without any purposeful targets, and therefore, relying on GPS sensor data for indoor data is not possible. The daily steps were measured using the FC4 built-in accelerometer sensor (i.e., GPS sensor is not used). They were advised with a set paragraph. “During the next 5 weeks, walk as much as you feel able. Any amount of walking is better than none. But please listen to your body and walk to a distance and pace level that you feel comfortable.*”* This paragraph was adopted in line with National Health Service advice for promoting walking among adults [29].

### Outcomes

In the absence of any direct guidance associated with the choice of key outcome measures on The COMET database (Core Outcome Measures in Effectiveness Trials; www.comet-initiative.org), the outcome measures selected here were streamlined from an earlier feasibility study conducted for such an intervention. During the baseline assessment, data were collected on gait, and hip-related disability using the Hip Disability and Osteoarthritis Outcome Score (HOOS) questionnaire [30]. The final assessment was carried out 5 weeks after the baseline appointment, and in addition to repetition of the baseline outcome measures, participants were also asked to complete the Psychosocial Impact of Assistive Devices Scale (PIADS) questionnaire [31]. Participants were also asked to keep a diary of their daily walking activities and the perceived intensity of their walk.

### Primary outcome measure

#### Walking activity

The walking activity was measured via the difference in the amount of daily walking pre- to postintervention as reported by the FC4 activity monitor. In the intervention group, this difference is assessed in terms of the amount of purposeful walking distance in kilometers, whereas in the control group the difference is based on daily step counts measured using the FC4 built-in accelerometer sensor. This data was downloaded by the lead researcher at the end of each week using the Fitbit app that was connected to the study’s Fitbit account.

### Secondary outcome measure

#### Gait analysis

**Fig. 2.**
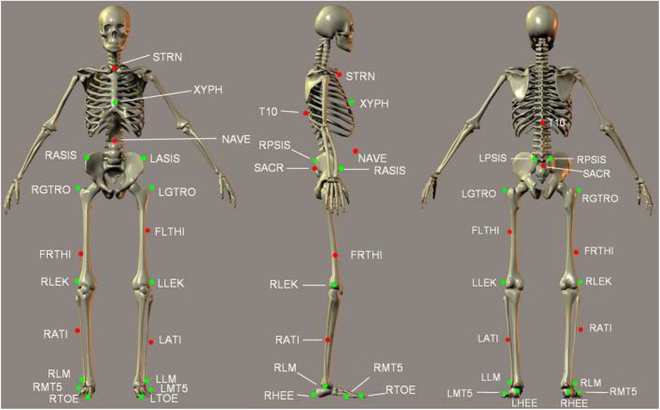
Diagram of markers used in HBM. T10, 10th thoracic vertebrae; SACR, sacrum bone; NAVE, navel; XYPH, xiphoid process; STRN, sternum; LASIS, pelvic bone left front; RASIS, pelvic bone right front; LPSIS, pelvic bone left back; RPSIS, pelvic bone right back; LGTRO, left greater trochanter of the femur; FLTHI, left thigh; LLEK, left lateral epicondyle of the knee; LATI, left anterior of the tibia; LLM, left lateral malleolus of the ankle; LHEE, left heel; LTOF, left toe; LMT5, left 5th meta tarsal; RGTRO, right greater trochanter of the femur; FRTHI, right thigh; RLEK, right lateral epicondyle of the knee; RATI, right anterior of the tibia; RLM, right lateral malleolus of the ankle; RHEE, right heel; RTOF, right toe; RMT5, right 5th meta tarsal.

The Gait Real-time Analysis Interactive Laboratory (GRAIL, Motekforce Link, Amsterdam, the Netherlands) system was used to carry out the gait analysis. GRAIL combined a fully instrumented treadmill with a self-paced option, as described by Sloot and van der Krogt [32]. The treadmill was feedback-controlled, which allowed participants to walk at their preferred speed. The gait analysis was carried out as per the protocol published on gait analysis using the GRAIL system [33]. Participants were fitted with 25 passive reflective markers using the human body model (HBM) lower body marker set [34]. Figure 2 shows the exact placements of all markers in the HBM lower body model. Following an acclimatization period, 3 sets of 25 gait cycles were recorded [33]. However, only spatiotemporal data (walking speed, cadence, and step length) were recorded for analysis. The reliability of the GRAIL system’s self-paced mode for walking speed has been previously documented [35], with the recommendation to capture a minimum of 23 gait cycles to accurately represent individuals’ walking characteristics [36]. Spatial-temporal gait parameters for all participants were exported in .CSV format and analyzed using MATLAB R2019b (The Mathworks Inc., USA). Gait analysis was chosen due to its demonstrated effectiveness in yielding objective insights into individual walking patterns and modalities before and after THR [37]).

### Patient reported outcome measures

#### Hip-related disability

Hip-related disability was assessed using the HOOS questionnaire [30]. The tool was validated in a sample of participants after THR surgery [38] and intended to be used to assess the individual’s opinion about their hip and associated problems and to evaluate symptoms and functional limitations related to the hip during their recovery process. To provide meaningful information to support the clinical effect of the 5-week programme on individuals, the minimal clinical important difference (MCID) for the HOOS was considered to be 24 [39].

#### Psychosocial impact of assistive devices scale

The PIADS was utilized to measure the effectiveness of the assistive device, in this case, the FC4 (e.g., all categories of assistive technology and not limited to any one type) on quality of life and sense of well-being [31,40]. This self-administered questionnaire is a valid and reliable tool in adults undergoing hip replacement surgery [41] and consists of 26 items, including 3 subscales (competence, adaptability, and self-esteem) [31]. Scores ranged from −3 (maximum negative impact) through zero (no perceived impact) to +3 (maximum positive impact).

### Qualitative outcomes

#### Activity diary

Participants were provided with an activity diary to record their daily walking activity. They were asked to record the distance walked in kilometers (km) or the number of steps taken, depending on the group they were randomized to. The activity diaries for both the intervention (Appendix S2) and control groups (Appendix S3) had a section where participants were able to document their feelings or reasons that may have affected their attempts to do their daily walk. For the content of the activity diary, we used content analysis [42]. The content of the activity diary was read line by line and coded by the lead researcher (S.B.), whereby meaning components were categorized. The content was further coded to interpret the meaning within their topic. These topics can be understood as the latent content of the text [42]. The purpose of this analysis was to explore the reasons why an individual was unable to perform their daily walk. However, the different topics were scrutinized for content that encompassed a reason beyond condition or feelings. Two topics (i.e., back to work and hobbies) were eligible for content analysis as the barriers to do a daily walk. Where appropriate, evidence from the activity diary was reported as a quotation to support the quantitative outcome measures.

The activity diary also included a quantitative section in which the participants were asked to rate the intensity of their daily walks using the Borg scale [43].

### Sample size

Twelve participants were chosen to take part in this pilot study. Six were randomized to the intervention group, and 6 were randomized to the control group. Given this was a pilot trial, a convenience sample size was selected, and a formal sample size calculation was not carried out.

### Randomization

The study used simple randomization. Each group in the study had 6 participants randomized to either the intervention or the control group, with a 1:1 allocation ratio. Randomization was done using a Sealed Envelope web-based system (reference number: 237466787579592) (https://www.sealedenvelope.com). The lead researcher undertook the randomization process and then informed participants of their group during the baseline visit.

### Statistical analysis

All data were analyzed using Microsoft Excel Version 2018 (Microsoft Corporation, 2022, retrieved from https://office.microsoft.com/excel). As this was a pilot study, all quantitative data (gait and patient reported outcome measures) were presented descriptively, using appropriate summary statistics. Given the differences in measurement units for the amount of walking completed by the intervention group (i.e., kilometers), and the control groups (i.e., steps), data were percentage normalized to the baseline walking levels. Due to the small sample size in each group, no statistical testing was completed. Within-group and between-group Cohen’s *d* effect sizes (44) were calculated for all variables having converted walking amount into percentage improvement. A sample size calculation to inform future studies was carried out using G*Power software (version 3.1.9.2).

## Results

### Recruitment

**Fig. 3.**
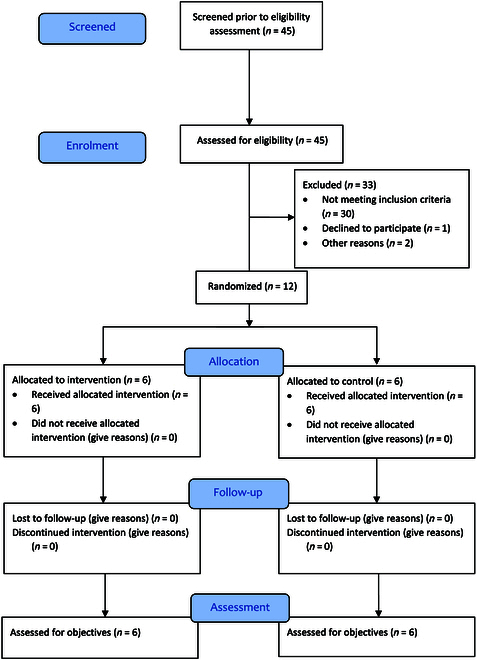
Participant flow diagram.

The participants’ flow diagram (Fig. 3) outlines the number of participants who contacted the lead researcher over a period of approximately 8 weeks, were assessed for eligibility, went through the randomization process, and were assessed.

### Participant demographics

Twelve adults were recruited to take part in this study. Tables 2 and 3 summarize the participant’s demographic information for the intervention and the control group respectively. The trial was completed by all participants, and there were no missing data. On average, the data on age, body mass index (BMI), and months postoperation were similar for both the intervention and the control group.

**Table 2.** Participants’ demographics information in the intervention group

Intervention group						
ID	Months post op	Age	Height (cm)	Weight (kg)	BMI (kg/m^2^)	Gender
I01	3	64.00	171.10	88.40	30.20	Female
I02	5	77.00	165.30	82.60	30.23	Male
I03	5	70.00	178.00	115.60	36.49	Male
I04	5	66.00	182.30	101.40	30.51	Male
I05	3	60.00	182.00	108.90	32.88	Male
I06	4	73.00	161.20	85.10	32.75	Female
Mean	4.17	68.33	173.32	97.00	32.17	
SD	0.98	6.22	8.88	13.64	2.44	

**Table 3.** Participants’ demographics information in the control group

Control group						
ID	Months post op	Age	Height (cm)	Weight (kg)	BMI (kg/m^2^)	Gender
C01	5	76.00	173.50	79.60	26.44	Male
C02	5	77.00	174.00	116.40	38.45	Male
C03	4	72.00	172.50	100.40	33.74	Male
C04	4	75.00	169.00	65.60	22.97	Female
C05	4	60.00	166.50	100.60	36.29	Female
C06	6	66.00	180.00	102.40	31.60	Male
Mean	4.67	71.00	172.58	94.17	31.58	
SD	0.82	6.69	4.64	18.28	5.91	

### Activity monitor

**Fig. 4.**
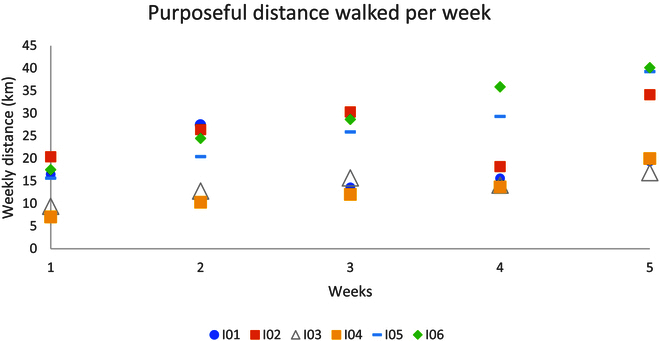
The total amount of purposeful distance walked by each participant per week.

**Fig. 5.**
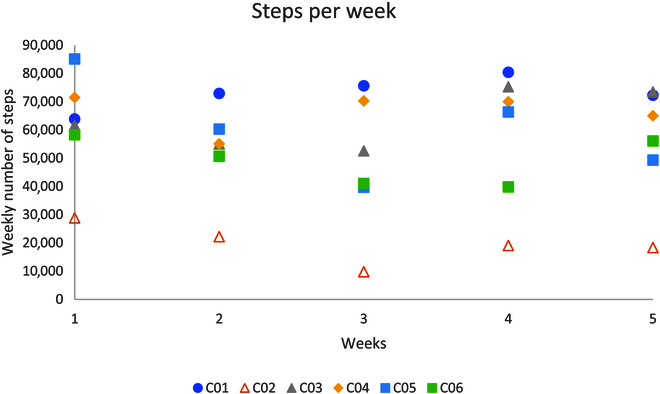
The total amount of steps taken by each participant per week.

Figures 4 and 5 outline individuals’ weekly total purposeful walk and step count for the intervention and the control group respectively. Participants I04, I05, and I06 achieved all of their weekly targets. Participants I02 and I03 managed to achieve 5 out of 6 targets. Participant I01 achieved 3 out of 6 weeks of their targets. All participants increased their baseline (week 1) purposeful walking distance amount with participant I01 having the lowest percentage (66.3%) and participant I04 having the highest percentage increase (183.8%).

In the control group, C01 and C03 increased their baseline (week 1) weekly steps by 25.9% and 22.1% respectively by the end of week 5; however, all other participants did not achieve more steps in the weeks after the baseline week.

### Gait analysis

**Fig. 6.**
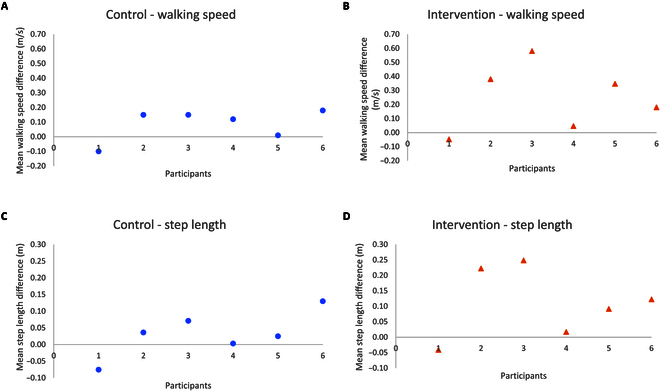
Mean difference in gait data for each participant in the intervention and the control group. (A) Mean difference in walking speed for each participant in the control group. (B) Mean difference in walking speed for each participant in the intervention group. (C) Mean difference in the step length of the operated side for each participant in the control group. (D) Mean difference in the step length of the operated side for each participant in the intervention group. (E) Mean difference in the cadence for each participant in the control group. (F) Mean difference in the cadence for each participant in the intervention group.

Figure 6 outlines individuals’ mean difference from pre to post intervention for the walking speed, step length of the operated side, and cadence of the intervention and the control group. Except for participant I01, and cadence data on participant I02, the walking speed, step length, and cadence increased across all other participants in the intervention group.

### Hip Disability and Osteoarthritis Outcome Score

**Fig. 7.**
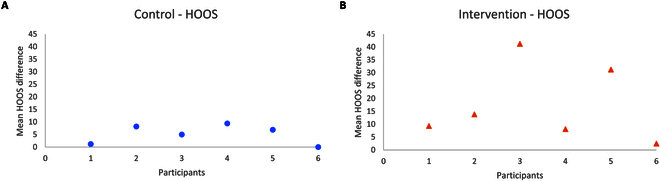
HOOS data for each participant in the intervention and the control group. (A) Mean difference in HOOS for each participant in the control group. (B) Mean difference in HOOS for each participant in the intervention group.

Figure 7 shows the data related to HOOS subjective mean score difference from pre to post intervention for the intervention and the control group. The MCID for pre to post-intervention was not seen in the HOOS score in any of the participants in the control group. However, a change beyond the MCID was seen in the HOOS outcomes, 41.2 and 31.2, for participants I03 and I04, respectively.

### Effect sizes

Table 4 shows the Cohen’s effect size (*d*) for the normalized walking amount, gait, and HOOS data.

**Table 4.** Within-group and between-group mean difference (pre to post intervention) (M_D_), standard deviation (SD), and the Cohen’s effect size (*d*)

	Intervention			Control			Between group
	M_D_	SD	*d*	M_D_	SD	*d*	*d*
Walking	104.68	60.98	1.72	-9.80	25.08	-0.39	1.27
Step length (m)	0.11	0.11	0.98	0.03	0.07	0.47	0.87
Walking speed (m/s)	0.25	0.23	1.06	0.09	0.11	0.79	0.89
Cadence (stride/min)	2.96	5.53	0.54	3.80	5.83	0.65	-0.15
HOOS	17.68	15.12	1.17	5.12	3.81	1.34	1.14

### The PIADS

Tables 5 and 6 show the PIADS scores for the intervention and control groups, respectively. The PIADS subscale for competence, and self-esteem, were better in the intervention group by more than 50%, in contrast to the control group. The adaptability score was 39% more positive for the intervention group in contrast to the control group.

**Table 5.** The PIADS scores for the intervention group

	Intervention		
ID	Competence	Adaptability	Self-esteem
I01	1.75	1.83	0.88
I02	2.55	3.00	2.50
I03	2.17	2.33	1.25
I04	1.45	2.17	1.38
I05	1.8	2.67	1.88
I06	2.64	3.00	2.13
Mean	2.07	2.50	1.67
SD	0.47	0.47	0.60

**Table 6.** The PIADS scores for the control group

	Control		
ID	Competence	Adaptability	Self-esteem
C01	0.33	0.83	0.63
C02	0.17	0.17	0.00
C03	1.18	2.00	0.75
C04	1.67	2.00	1.38
C05	1.55	3.00	1.00
C06	1.08	1.17	0.88
Mean	1.00	1.53	0.77
SD	0.62	1.01	0.46

### Ancillary analyses of sample size

A sample size calculation was carried out for walking distance based on the effect size of 1.27 from this pilot study, with alpha at 0.05 and power at 90%, a sample size of 24 is required.

### Activity diary

The walking intensity of participants in either the intervention or the control group, reported through the Borg scale, did not exceed the moderate activity level for the duration of the 5 weeks. The main theme derived through analysis of the activity diary for the intervention group was the “enjoyment” of walking outdoors and “exceeding expectations” (i.e., going beyond the level they felt capable of). Other factors beyond the condition or feelings were outlined by individual participants and were explored further. For example, I01 returned to work from week 3 onwards doing a daily 8 hours shift in a supermarket. Furthermore, she suffered from left knee pain:

“Started back at work today after 14 weeks off, 7297 steps at work, couldn’t manage a long dog walk, hip felt like it had done enough and left knee hurting”*.*

Similarly, participant I02, was a keen fisherman and on week 4 he returned to his usual long fishing sessions. He camped by a river for the entire week and some of the fishing sessions were a full day’s activity:

“Went back fishing, not much walking today. Hip joint gets stiff when sitting for a long length of time. Got up walking about for a few minutes and it got easier.”

The main themes found in the control group’s responses were “bad weather”, “felt down”, “not a good day”, “busy”, and “did my physio only today”. Exploring topics beyond the condition or feelings, showed “gardening” as a main theme among the control group as it was repeated 13 times on different occasions. Participant C01 did not report any condition or feeling that may have affected his walk.

## Discussion

This study was the first randomized trial to report the effect of the outdoor purposeful walk, monitored using a commercial activity monitor. The aim of this study was to determine the effect of an intervention where walking distance was used as a goal to increase daily walking activity using a commercially available activity monitor in people 3 to 6 months after THR surgery. It was our aim to compare this intervention group against a control group who reported their daily steps as opposed to a daily distance outdoor walk. No target on increasing step count was set. Our findings suggest that the purposeful walking intervention was successful in increasing daily walking activity and function in contrast to the control group.

Although commercial activity monitors in interventions to promote physical activity in the form of walking is a relatively new phenomenon, there has been a rapid increase in their popularity and use in research during the last decade [19,45]. However, when it comes to THR studies [20,21], the focus for monitoring or enhancing the amount of walking has been merely on the step count parameter. Despite some benefit in enhancing daily activity [21], the evidence shows that the counting step is not a stimulus for enhancing long-term functional and gait recovery in THR patients’ rehabilitation [46]. Additionally, reports have outlined the importance of individualized support and how an individual would appreciate a continuous personalized goal [6,47]. This is perhaps why the results from the control group in this study showed that despite full adherence to using the FC4, the number of steps decreased during the intervention. This is in line with findings from Ostlind et al. [48], where despite achieving up to 7,000 daily steps initially, over a period of 12 weeks, the number of steps taken by individuals with hip osteoarthritis decreased slightly, but gradually over time, in the absence of a personalized daily goal. Therefore, it can be suggested that an activity monitor may aid in the optimization of daily walking, but it is not a panacea and other factors such as goal setting could play a crucial role in enhancing daily walking activity. Goal setting could also provide a motivation [49] as there was evidence of low-level mood among the control group and repetition of themes such as “lazy day” and “felt down” was seen in 4 out of 6 participants in this group.

Age, BMI, and postsurgical period have previously been suggested as the factors associated with the level of activity post-THR surgery [20,50]. Fortunately, the average age, BMI and months post operation for the intervention and control groups were similar in this study. However, given there are currently no comparable data available on the average outdoor walking distance for individuals post-THR surgery, we compared our control group data to the Tang et al. [12] study that had participants with similar age and BMI, (that is 61.6 ± 10.2, BMI 25.5 ± 5.9). This study reported that at 3 months post operation, the THR participants did an average daily step of 4,526 ± 2,721. Another study reported a similar number of steps, 4,632 ± 2,246, in a group of 61-year-old Japanese females 6 months post-THR surgery [51]. Participants in this study exceeded these numbers and suggested that except for participant C02 (2,811 steps per day), participants in the control group took 7,090 ± 2,739 steps per day during the 5 weeks of the study. However, this number of steps is comparable with the data from the healthy population of the Tang et al. [12] with a similar age group. Our findings, in congruence with the literature, suggest that individuals may expect to return to the level of activity similar to the healthy matched aged group following THR as early as 3 months and may improve in the later postoperative periods (for example, 5 months and onward).

The gait parameters showed improvement for both the control and intervention groups. This is to be expected as participants gradually recover from their surgery regardless of their individualized rehabilitation programs. However, despite the lack of statistical analysis, on average, the mean walking speed improved by 0.09 ± 0.1 m/s in the control group in contrast to 0.25 ± 0.2 m/s in the intervention group. Furthermore, the step length of the operated leg was improved by 0.03 ± 0.06 m in the control group in contrast to 0.11 ± 0.1 m in the intervention group. Furthermore, as suggested by Cohen [44], effect sizes may be categorized as small (0.2), medium (0.5), and large (0.8). Except for the cadence, the effect size for all quantitative outcome measures were large. Given the effect size provides insight into the magnitude of the difference between groups, the large effect size observed here may act as an indicator that the findings from this study have practical significance. Therefore, it may be suggested that a purposeful walking intervention could be a more effective stimulus than step count in improving selected gait spatiotemporal parameters post-THR surgery. However, further studies with larger sample sizes and longer follow-up are needed to assess the evidence on the significance of the effect of the purposeful walk in contrast to step count.

Participants’ characteristics, hobbies, psychological feelings, and comorbidities influenced the level of activities in either group. Recognizing pain and discomfort elsewhere (e.g., knee) and extended factors such as returning to work at 3 months postsurgery reduced the amount of outdoor walking which was carried out by participant I01. The diary information suggested that on average participant I01 was doing over 7,000 steps per day during her indoor working hours. However, she could not continue with the progress she made during the first 3 weeks and was unable to achieve her daily outdoor purposeful walks. It could possibly be suggested that as participant I01 was the only participant who did not improve in any of her gait parameters, the lack of outdoor walks may have had an influence.

The average difference in HOOS outcome measures in the intervention group was 23.8 ± 14.9 (excluding 2 participants, I04 and I06, who score more than 90 in their baseline assessment) in contrast to the average difference of 6.14 ± 3.2 (excluding 1 participant, C06, who also scored more than 90 at the baseline) suggests that intervention had a bigger impact on the subjective self-perceived outcome measure.

Studies have reported that an ability to walk even a short distance outdoors can be meaningful for successful and independent living at home among the THR group as well as enhancing their physical function [17,52]. There was evidence of a greater psychological effect on participants within the intervention group with all subscales of PIADS showing greater improvement in contrast to subjective answers from the control group. Participants mainly saw the benefit of the FC4 and its GPS functionality upon seeing the maps of the routes they have walked. Meanwhile the outdoor walk provided a platform for further interactions, whether that was with their pets, friends, family, or even members of the public during their daily walks. This is important, as current evidence suggests that majority of THR patients feel socially isolated even at 12 months post their surgery [53].

The limitations in this study are mainly inherent to the study methodology. There was no formal power calculation and therefore the sample size in each group was too small for other than minimal statistical analysis. However, we strengthened our methodology by adopting the randomization process for assigning the study participants to each group. The study had an additional limitation regarding the comparison of metrics used to measure daily activity. The intervention group’s daily activity was assessed based on walking distance, while the control group’s activity was measured by step count. This discrepancy in measurement methods raises concerns about the fairness of directly comparing the 2 groups. To establish a more robust basis for evaluating the impact of FC4 on daily activity, it would be advantageous to include both walking distance and step count metrics for both groups in future studies. This approach would offer more substantial evidence for comparing the effects of FC4 on daily activity. Acknowledging the impact of other movements is equally vital. To address this, we integrated activity diaries as a central element of our methodology. These diaries encouraged participants to personally categorize or input their activity type when engaging in nonstandard actions. This intentional incorporation served a dual objective: not only did it enhance precision, but it also empowered users to provide contextual insights.

Moreover, the participants recruited in our study had their THR completed by different surgeons using different techniques and surgical approaches, which may influence their early postoperative recovery time [54]. To address this limitation, we included participants who were at least 3 months postoperation and could confirm they are discharged from their surgical care. Additionally, studies suggest that regardless of surgical approach or technique, at 3 months post-THR surgery, patients are ready to return to their normal activity [55].

Unfortunately, studies have suggested that despite precision, the FC4 is not accurate in slow-walking participants [56,57]. Therefore, when it came to our analysis of mean changes, the effect size, and also sample size calculation, the data should be approached with caution. The effect size was large and therefore sample size calculation may be underpowered with only 24 samples per group. Additionally, there was a wide spread of data across both the control and intervention group for daily walking activity. Moreover, the implementation of a personalized plan for each participant ensured the continuation of any inherent device discrepancies, as the same device remained in use throughout the entire 5-week intervention period. Thus, we reported individual data as well as an average across all outcome measures to provide more comprehensive access to the outcomes. Finally, a follow-up period of 5 weeks may be too short to assess any notable changes in our study outcomes, as other studies have shown improvement in physical activity with a longer follow-up time [11,58].

In a randomized controlled trial, participants who received the purposeful intervention using a commercial activity monitor with a daily outdoor distance goal had higher activity levels after THR, compared to participants who were in the control group and reported daily step counts. The data for gait, HOOS, and PIADS appeared to be better in the intervention group in contrast to the control group. However, further research with a larger sample size is required to provide tangible evidence on the significance of the effect of the purposeful walk in contrast to step count.

## Data Availability

The datasets generated during and/or analyzed during the current study are available from the corresponding author on reasonable request.
