# Supplementary material for: Can a Purposeful Walk Intervention with a Distance Goal Using an Activity Monitor Improve Individuals’ Daily Activity and Function Post Total Hip Replacement Surgery. A Randomized Pilot Trial
Source: Cyborg Bionic Syst. 2023 Nov 30;4:0069. doi: 10.34133/cbsystems.0069 (PMC10907016; doi:10.34133/cbsystems.0069)
Supplement: Supplementary 1 — Appendix S1 to S3 [file cbsystems.0069.f1.zip › renamed_951c7.docx]

## **Activity diary (Intervention)**

**NAME**…………………………………………………………………………………………….

Please use this diary to record your daily activity starting from week 2. We would like you to record the amount of distance you walked in kilometres (Km) as recorded on your activity monitor and how intensely you felt you were exercising on average (Borg Scale). The Borg Scale is provided for you below, please use this as a guide and score yourself out of 10, (e.g. 1/10 representing hardly any exertion and 10/10 representing maximum effort).

Bring your activity diary with you to your follow-up assessment for review by the research team.


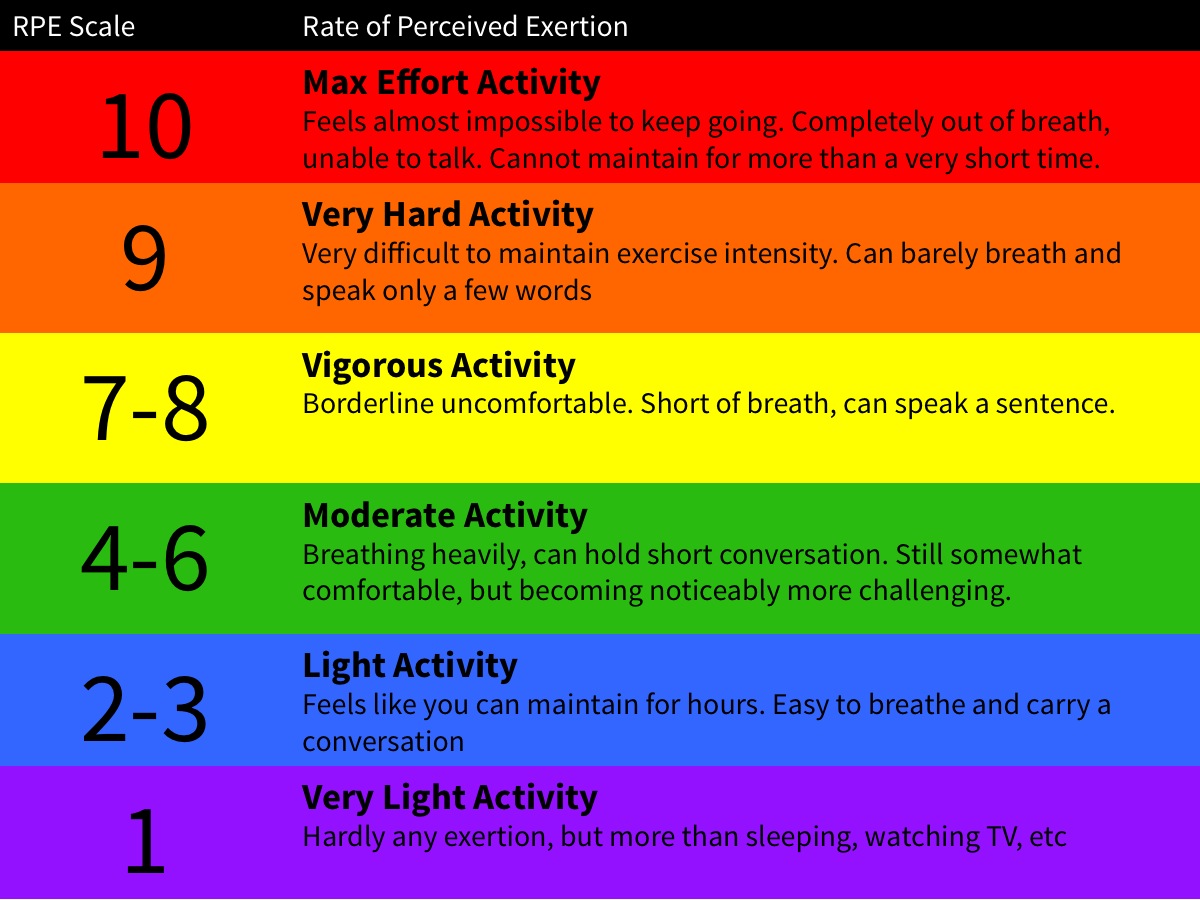


| **Date**  ***(Please write the date for every entry below)*** | ***DO NOT WRITE ANYTHING HERE*** | Daily Walk | Please use this section to expand on any condition/feelings which may have affected your daily outdoor walk for this day |
| --- | --- | --- | --- |
|  | Total Distance *(Km)* |  |  |
|  | Intensity *(Borg Scale i.e. 4/10)* |  |  |

|  | Total Distance *(km)* |  |  |
| --- | --- | --- | --- |
|  | Intensity *(Borg Scale i.e. 4/10)* |  |  |
|  | Total Distance *(Km)* |  |  |
|  | Intensity *(Borg Scale i.e. 4/10)* |  |  |
|  | Total Distance *(Km)* |  |  |
|  | Intensity *(Borg Scale i.e. 4/10)* |  |  |
|  | Total Distance *(Km)* |  |  |
|  | Intensity *(Borg Scale i.e. 4/10)* |  |  |

|  | Total Distance *(Km)* |  |  |
| --- | --- | --- | --- |
|  | Intensity *(Borg Scale i.e. 4/10)* |  |  |
|  | Total Distance *(km)* |  |  |
|  | Intensity *(Borg Scale i.e. 4/10)* |  |  |
|  | Total Distance *(Km)* |  |  |
|  | Intensity *(Borg Scale i.e. 4/10)* |  |  |
|  | Total Distance *(Km)* |  |  |
|  | Intensity *(Borg Scale i.e. 4/10)* |  |  |
|  | Total Distance *(Km)* |  |  |
|  | Intensity *(Borg Scale i.e. 4/10)* |  |  |
|  | Total Distance *(Km)* |  |  |
|  | Intensity *(Borg Scale i.e. 4/10)* |  |  |
|  | Total Distance *(km)* |  |  |
|  | Intensity *(Borg Scale i.e. 4/10)* |  |  |
|  | Total Distance *(Km)* |  |  |
|  | Intensity *(Borg Scale i.e. 4/10)* |  |  |
|  | Total Distance *(Km)* |  |  |
|  | Intensity *(Borg Scale i.e. 4/10)* |  |  |
|  | Total Distance *(Km)* |  |  |
|  | Intensity *(Borg Scale i.e. 4/10)* |  |  |
|  | Total Distance *(Km)* |  |  |
|  | Intensity *(Borg Scale i.e. 4/10)* |  |  |
|  | Total Distance *(km)* |  |  |
|  | Intensity *(Borg Scale i.e. 4/10)* |  |  |
|  | Total Distance *(Km)* |  |  |
|  | Intensity *(Borg Scale i.e. 4/10)* |  |  |
|  | Total Distance *(Km)* |  |  |
|  | Intensity *(Borg Scale i.e. 4/10)* |  |  |
|  | Total Distance *(km)* |  |  |
|  | Intensity *(Borg Scale i.e. 4/10)* |  |  |
|  | Total Distance *(Km)* |  |  |
|  | Intensity *(Borg Scale i.e. 4/10)* |  |  |
|  | Total Distance *(Km)* |  |  |
|  | Intensity *(Borg Scale i.e. 4/10)* |  |  |
|  | Total Distance *(Km)* |  |  |
|  | Intensity *(Borg Scale i.e. 4/10)* |  |  |
|  | Total Distance *(Km)* |  |  |
|  | Intensity *(Borg Scale i.e. 4/10)* |  |  |
|  | Total Distance *(km)* |  |  |
|  | Intensity *(Borg Scale i.e. 4/10)* |  |  |
|  | Total Distance *(Km)* |  |  |
|  | Intensity *(Borg Scale i.e. 4/10)* |  |  |
|  | Total Distance *(Km)* |  |  |
|  | Intensity *(Borg Scale i.e. 4/10)* |  |  |
|  | Total Distance *(Km)* |  |  |
|  | Intensity *(Borg Scale i.e. 4/10)* |  |  |
